# Cell-Cell Interaction-Mediated Signaling in the Testis Induces Reproductive Dysfunction—Lesson from the Toxicant/Pharmaceutical Models

**DOI:** 10.3390/cells11040591

**Published:** 2022-02-09

**Authors:** Lingling Wang, Tiao Bu, Xiaolong Wu, Sheng Gao, Xinyao Li, Angela Bryanne De Jesus, Chris K. C. Wong, Hao Chen, Nancy P. Y. Chung, Fei Sun, C. Yan Cheng

**Affiliations:** 1 Department of Urology and Andrology, Sir Run Run Shaw Hospital, Zhejiang University School of Medicine, Hangzhou 310016, China; wangll500@126.com (L.W.); tiao-bu@foxmail.com (T.B.); wuxiaolong19900217@163.com (X.W.); gses1969@163.com (S.G.); 2 Institute of Reproductive Medicine, Nantong University School of Medicine, Nantong 226001, China; lixy1997sh@gmail.com (X.L.); chenhao@ntu.edu.cn (H.C.); 3 Department of Biology, Nyack College, New York, NY 10004, USA; dejesusa@nyack.edu; 4 Department of Biology, Croucher Institute for Environmental Sciences, Hong Kong Baptist University, Hong Kong, China; ckcwong@hkbu.edu.hk; 5 Department of Genetic Medicine, Cornell Medical College, New York, NY 10065, USA; puc2001@med.cornell.edu; 6 The Mary M. Wohlford Laboratory for Male Contraceptive Research, Center for Biomedical Research, Population Council, 1230 York Avenue, New York, NY 10065, USA

**Keywords:** testis, toxicants, cadmium, PFOS, adjudin, cell-cell interactions, signaling proteins

## Abstract

Emerging evidence has shown that cell-cell interactions between testicular cells, in particular at the Sertoli cell-cell and Sertoli-germ cell interface, are crucial to support spermatogenesis. The unique ultrastructures that support cell-cell interactions in the testis are the basal ES (ectoplasmic specialization) and the apical ES. The basal ES is found between adjacent Sertoli cells near the basement membrane that also constitute the blood-testis barrier (BTB). The apical ES is restrictively expressed at the Sertoli-spermatid contact site in the apical (adluminal) compartment of the seminiferous epithelium. These ultrastructures are present in both rodent and human testes, but the majority of studies found in the literature were done in rodent testes. As such, our discussion herein, unless otherwise specified, is focused on studies in testes of adult rats. Studies have shown that the testicular cell-cell interactions crucial to support spermatogenesis are mediated through distinctive signaling proteins and pathways, most notably involving FAK, Akt1/2 and Cdc42 GTPase. Thus, manipulation of some of these signaling proteins, such as FAK, through the use of phosphomimetic mutants for overexpression in Sertoli cell epithelium in vitro or in the testis in vivo, making FAK either constitutively active or inactive, we can modify the outcome of spermatogenesis. For instance, using the toxicant-induced Sertoli cell or testis injury in rats as study models, we can either block or rescue toxicant-induced infertility through overexpression of p-FAK-Y397 or p-FAK-Y407 (and their mutants), including the use of specific activator(s) of the involved signaling proteins against pAkt1/2. These findings thus illustrate that a potential therapeutic approach can be developed to manage toxicant-induced male reproductive dysfunction. In this review, we critically evaluate these recent findings, highlighting the direction for future investigations by bringing the laboratory-based research through a translation path to clinical investigations.

## 1. Introduction

Toxicants that were shown to exert their disruptive effects at the intercellular junctions in target organs, including cells and tissues, such as at the neuromuscular interface, between embryonic mesenchymal cells during development possibly to perturb intercellular communication, were first reported in the 1950s and 1980s [1,2,3,4]. Interestingly, toxicants that exert their disruptive effects at the cell-cell interface, most notably in cell junctions (or cell adhesion sites) in testicular cells, such as Sertoli cells, germ cells, and Leydig cells, which in turn lead to defects in spermatogenesis and male reproductive dysfunction, were not found in the literature until the 1980s and 1990s [5,6,7,8]. Since then, studies that evaluate toxicant-induced changes in cell-cell interactions in the testis, the epididymis and the prostate, which in turn impede male reproductive function, leading to male infertility, including cadmium, PFOS (perfluorooctane sulfonate), phthalates, and others, have grown rapidly, and many of these studies have been summarized in recent reviews [9,10,11,12,13,14,15,16,17,18] (Table 1). Since many of these intercellular junctions utilize actin as attachment sites for the integral membrane proteins and/or adaptors of the adhesion protein complexes, it is not unexpected that cytoskeletons, such as actin and microtubule cytoskeletons, are one of the primary targets of toxicants, as recently reviewed [16,19,20]. Furthermore, the most notable and consistent phenotype in these earlier studies following exposure of rodents to toxicants is germ cell exfoliation from the seminiferous epithelium. Nonetheless, the signaling proteins and the detailed signaling cascade utilized by toxicants to mediate male reproductive dysfunction through changes at the cell junction level remain largely unexplored. Even though earlier studies that focused on studying toxicant-induced germ cell apoptosis have illustrated the involvement of the Fas system [21,22,23,24] or the ion channel (e.g., calcium ion channel), and they are likely key pathways of cell apoptosis in the mammalian testis [25,26,27]. Nevertheless, the involved signaling proteins and the pathways at the cell junction level remain unknown.

Studies in recent years, however, have reported the involvement of members of the mitogen-activated protein kinases (MAPKs), such as p38 MAPK and ERK1/2 and their activated isoforms, in mediating blood-testis barrier (BTB) function at the Sertoli cell-cell interface [28,29,30,31,32]. In this context, it is of interest to note that the BTB is constituted by the actin-based tight junction (TJ), basal ES (ectoplasmic specialization) and gap junction, as well as the intermediate filament-based desmosome [33,34,35]. The BTB, in turn, divides the seminiferous epithelium into the basal and apical (adluminal) compartments. These reports have provided the initial indication that intercellular junctions in the testis may be one of the targets of toxicants. In fact, one of the earliest studies illustrating the likely involvement of MAPKs in mediating toxicant (e.g., cadmium)-induced Sertoli cell TJ barrier function at the BTB in vivo was first reported in 2003 [32], and also in vitro [31]. Subsequent studies using Sertoli cell cultures or Sertoli-germ cell cocultures have also reported the involvement of other signaling proteins (e.g., ROCK, LIMK) and small GTPases (e.g., Rho B), besides MAPKs (e.g., p38 MAPK), in toxicant-induced testis and Sertoli cell injury [31,36]. In short, studies have shown that PFOS and cadmium are two of the environmental toxicants, among others, that induce Sertoli cell and/or testis injury by perturbing cell-cell interactions in the testis through activation of MAPKs, including p38-MAPK, ERK1/2 and JNK [32,37,38,39]. It is also noteworthy to mention that the doses of toxicants (e.g., cadmium chloride at 3 mg/kg b.w. in studies in vivo or 5–10 µM in primary Sertoli cell cultures in vitro, PFOS at 20 µM in primary Sertoli cell cultures in vitro) used in these studies were not cytotoxic to the testicular cells [40,41]. However, these doses were higher than normal human exposures. For instance, the current oral TWI (tolerable weekly intake) for cadmium is 2.5 µg/kg b.w. (http://www.efsa.europa.eu/en/efsajournal/pub/1975, accessed on 7 December 2021) and the oral TDI (tolerable daily intake) for PFOS is 150 ng/kg/day (https://www.efsa.europa.eu/en/news/efsa-opinion-two-environmental-pollutants-pfos-and-pfoa-present-food, accessed on 7 December 2021) for humans [42]. Thus, these levels are considerably lower than the doses used for acute-dose studies in rodents in order to yield distinctive phenotypes within a short experimental period. However, cadmium and PFOS have a relatively long human elimination half-life of >20 years and 5 years, respectively [42], and high levels of these toxicants can build up in the human body, especially among industrial workers. Collectively, these findings thus provide an opportunity to manage toxicant-induced male reproductive dysfunction. For instance, if the signaling protein(s) that are responsible for mediating the effects of toxicant-induced reproductive dysfunction is known, the use of specific inhibitors and/or activators, along with agonists and/or antagonists, can be explored for their use to block, and perhaps rescue (or reverse), toxicant-induced male Sertoli cell (or testis) injury or germ cell exfoliation. This possibility has been examined in recent studies, and these reports are carefully evaluated below. In brief, these findings thus open a new window to manage male reproductive dysfunction. However, since the involvement of MAPKs, ERKs and JNKs (and also their role in oxidative stress) in mediating toxicant-induced male reproductive dysfunction has recently been reviewed [43,44,45,46], we do not discuss these MAPK-based signaling proteins in this short review to avoid redundancy.

**Figure 1:**
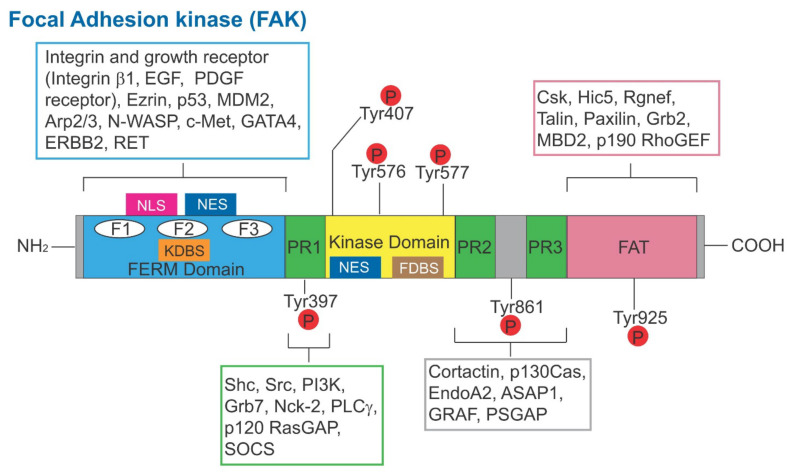
Schematic illustration of the functional domains of human FAK. The human FAK is a polypeptide comprised of 1058 amino acid residues. From its N-terminus, it is comprised of the FERM domain, to be followed by the intrinsic kinase domain and the FAT domain at its *C*-terminus. It has three distinctive PR1 domains and several distinctive Tyr phosphorylation sites. Within the FERM domain, it also consists of NLS, NES, KDBS and F1-F3 domains. The intrinsic kinase domain also consists of the NES and FDBS domains. Abbreviations used: FERM, F for 4.1 protein, E for ezrin, R for radixin and M for moesin; NLS, nuclear localization sequence; KDBS, kinase domain binding site; NES, nuclear export sequence; FDBS, FERM domain binding site; EGF, epidermal growth factor; PDGF, platelet-derived growth factor; p53, tumor protein p53; Mdm2, mouse double minute 2 homology (also known as E3 ubiquitin-protein ligase, a regulator of the p53 tumor suppressor); Arp2/3, actin-related protein 2/3 complex; N-WASP, neuronal Wiskott–Aldrich syndrome protein; c-Met, MET proto-oncogene, receptor tyrosine kinase; GATA4, GATA binding protein 4; ERBB2, Rrb-b2 receptor tyrosine kinase 2; RET, rearranged during transfction, a proto-oncogene; Shc, SHC-adaptor protein; Src, cellular Src transforming kinase; PI3K, phosphatidylinositol 3-kinase; Grb7, growth factor receptor bound protein 7; Nck-2, NCK adaptor protein 2; PLCγ, phospholipase C γ1; p120 RasGAP, RAS p21 protein activator 1; SOCS, suppressor of cytokine signaling 3; p130Cas, p130 Cas family acaffolding protein; ASAP1, ArfGAP with SH3 domain, ankyrin repeat and PH domain 1; GRAF, GTPase regulator associated with FAK; PSGAP, a novel pleckstrin homology and Src homology 3 domain containing RhoGAP protein; Csk, *C*-terminal Src kinase; Hic5, transforming growth factor ß1 induced transcript 1; Rgnef, Rho guanine nucleotide exchange factor 28; Grb2, growth factor receptor bound protein 2; MBD2, MBD2, methyl-CpG binding domain protein 2; p190 RhoGEF, an activator of Rho-family GTPases.

**Figure 2:**
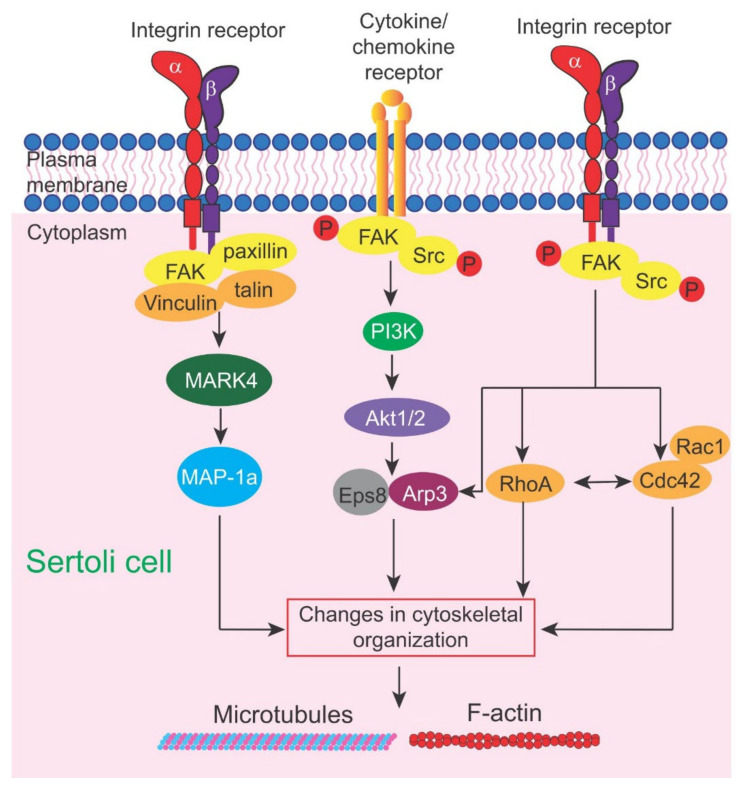
Schematic illustration of the signaling cascade and the involving protein kinases and regulatory biomolecules that mediate FAK-based signaling to support spermatogenesis. This figure was prepared based on current findings in the field, as discussed herein (see text for details). MARK4, microtubule-affinity regulating kinase 4; MAP-1a, microtubule-associated protein 1a.

**Figure 3:**
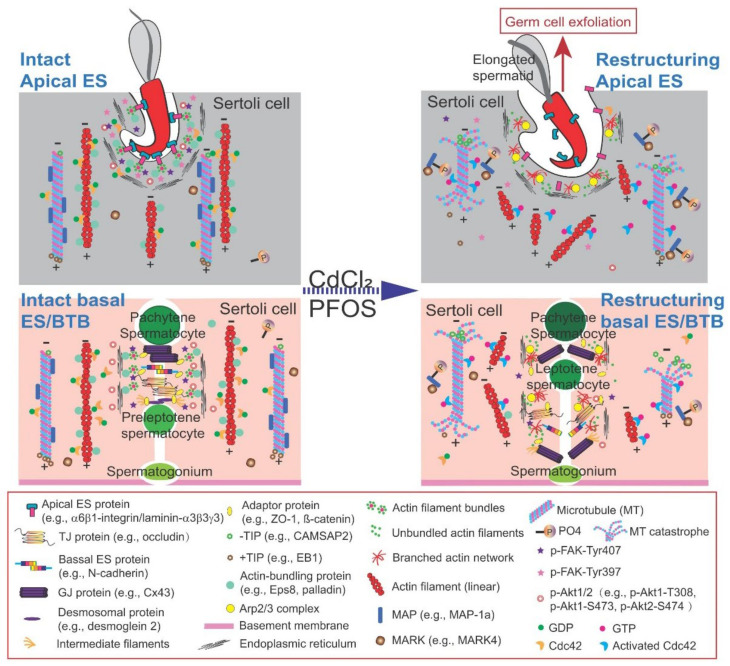
Schematic illustration of the current working model of the FAK-based signaling involving Cdc42 that modulates remodeling of apical ES and basal ES/BTB to support spermatogenesis. The apical ES (top) and basal ES/BTB (lower) shown on the left panel illustrate the intact ES at the Sertoli-spermatid and Sertoli cell-cell interface, respectively, such as at stage VII of the epithelial cycle. However, treatment of rats or Sertoli cells cultured in vitro or in the testis in vivo with CdCl_2_ or PFOS based on studies discussed herein have shown that these toxicants induced Sertoli cell and/or testis injury by inducing remodeling of the ES at both sites. In brief, for the MT cytoskeleton, there is a change in the distribution of MT regulatory proteins, such that + TIP (e.g., EB1) is no longer tightly bound to the MT plus (+) end, with a concomitant increase in the binding of -TIP (e.g., CAMSAP2), which in turn de-stabilize the MTs, facilitating MT catastrophe. On the other hand, MAPs (e.g., MAP1a) no longer tightly bind onto the MTs to stabilize the MT cytoskeleton. Instead, MARK4 induces phosphorylation of MAPs, causing their detachment from microtubules, which also de-stabilizes MTs, leading to MT catastrophe. For the actin cytoskeleton, there is an increase in the Arp2/3 complex activity through induction of its upstream regulator (e.g., N-WASP), causing branched actin polymerization. On the other hand, there is a considerable decline in the expression of actin-bundling protein (e.g., palladin) or the actin barbed end-capping and bundling protein Eps8. This reduced actin-bundling activity, coupled with an increase in Arp2/3 complex activity, lead to remodeling of the F-actin network, facilitating the conversion of actin filaments from a bundled to an unbundled configuration, thereby de-stabilizing the F-actin network. These changes thus contribute to reduced adhesion at the Sertoli-spermatid interface and the Sertoli cell-cell interface at the apical ES and basal ES/BTB, respectively. In brief, exfoliation of elongated spermatids and unwanted remodeling of the BTB take place simultaneously, causing defects in spermatogenesis that lead to male reproductive dysfunction.

Instead, we focus our discussion on the latest findings regarding the role of an emerging signaling protein and its downstream pathway(s) in mediating toxicant-induced Sertoli and testis injury based on studies in rodents and humans, namely the focal adhesion kinase (FAK) (Figure 1), the Akt1/2 (Figure 2) and the FAK/Cdc42-based signaling pathways (Figure 2). It is rather unusual that FAK plays such an important role in mediating cell-cell interaction in the testis since focal adhesion kinase (FAK), as its name implies, is a signaling protein involved in focal adhesion complex (FAC) dynamics. FAK is one of the best-studied cytoplasmic non-receptor protein tyrosine kinases [47,48]. It is restrictively expressed at the actin-based cell-extracellular matrix (ECM) interface designated FAC (or focal contact) in multiple epithelia and/or endothelia (and a cell-matrix anchoring junction type), but not at the cell-cell interface [49]. Interestingly, in the testis, studies using electron microscopy have shown that there is no ultrastructure similar to FAC detected at the base of the seminiferous epithelium between Sertoli cells and the basement membrane (a modified form of ECM in the testis) [50,51], which is the site where FAK supposed to exert its regulatory function. Instead, FAK and two of its activated/phosphorylated forms, p-FAK-Y397 and p-FAK-Y407, are robustly but restrictively expressed at the apical ES [52,53] and apical/basal ES [54], respectively, which are the testis-specific cell-cell anchoring junction type [35,55,56] (Figure 3). Critical evaluation of these data, and, in particular, findings from more recent reports, have shed new insights regarding the path that should be taken so that this information can be considerably expanded in future studies. One of the main goals is to bring this research to a translation path so that this information can be brought to clinics. It is noted that our discussion in this review relies mostly on findings derived from studies in the rat testes unless otherwise specified. However, studies based on scRNA-Seq have shown that many of the proteins found in the rat testes, including those regulatory proteins residing at the apical and basal ES, are also expressed in human testes [57], implicating that our evaluation here is applicable to human spermatogenesis.

## 2. Unique Features of Cell Junctions in the Testis

Studies based on the use of in vivo and in vitro models have shown that cell junctions at the Sertoli cell-cell or Sertoli-germ cell interface in the testis are one of the targets of environmental toxicants, including cadmium, PFOS, bisphenol A (BPA), phthalates and others [8,31,32,36,37,40,58,59,60,61]. Interestingly, intercellular junctions in the testis share many features of epithelial junctions in other tissues/organs, while there are some unique features in the testis not found in other tissues/organs. For instance, in the rat testis, at the Sertoli-spermatid (steps 8–19 spermatids) interface and the Sertoli cell-cell interface, there is a unique cell-cell anchoring junction known as the apical ES and basal ES, respectively [35,56,62] (Figure 3). Both apical and basal ES share similar ultrastructural features in which a distinctive array of actin filament bundles that are aligned perpendicular to the plasma membranes at either the Sertoli-spermatid (apical ES) or the Sertoli cell-cell (basal ES) interface is found (Figure 3). This array of actin bundles is sandwiched in between the apposing plasma membranes of Sertoli cell-spermatid (apical ES) or Sertoli cell-cell (basal ES) and the cisternae of endoplasmic reticulum (ER) in Sertoli cells at the ES, and is not found in spermatids (Figure 3). The apical and basal ES, in turn, are supported by another network of actin filaments and protofilaments of microtubules that are aligned parallel to the plasma membrane that lay adjacent to the actin filament bundles (Figure 3), illustrating the intimate structural relationship between the actin and microtubule cytoskeletons. These networks of actin and MT cytoskeletons thus confer ES with unusual adhesive strength. As such, ES is considered to be one of the strongest adhesive cell-cell junctions in the mammalian body based on a study in which the force required to “pull” the involving Sertoli cell and spermatid apart was quantified [63]. Interestingly, once apical ES appears between haploid step 8 spermatids and Sertoli cells in stage VII tubules of rat testes, it is the only anchoring junction between developing spermatids and Sertoli cells, replacing the desmosome and gap junctions, and persists until the end of spermiogenesis in the rat testis (Figure 3). Apical ES undergoes degeneration in late stage VIII tubules to facilitate the release of sperm at spermiation (Figure 3). Interestingly, unlike apical ES, the basal ES does not exist alone. Instead, it coexists with the actin-based TJs and gap junctions, which, together with the intermediate filament-based desmosome, they constitute the BTB in the testis [34,35]. As such, these junctions work in concert as a group to confer strong adhesion between adjacent Sertoli cells near the base of the seminiferous epithelium at the BTB, making the BTB one of the tightest blood-tissue barriers in the mammalian body, similar to the blood-brain barrier (BBB) [34,64,65,66,67]. Even though the ES is such a strong adhesive junction, it is exceedingly sensitive to toxicants, in particular the pharmaceutical drug adjudin [15,62,68]. Indeed, studies have shown that adjudin (a male contraceptive drug under intense investigation in our laboratory [69,70,71]) exerts its effects primarily at the actin cytoskeleton [72], and it effectively perturbs apical ES adhesion [68]. Other studies have also shown that the basal ES/BTB and the apical ES are also highly sensitive to the environmental toxicant cadmium in the testis [61,73,74]. These findings seemingly suggest that the ES that supports spermatid and Sertoli cell adhesion in the seminiferous epithelium during spermatogenesis may be utilizing specific signaling proteins and/or cascades, perhaps different from other cell epithelia. If these signaling proteins and/or pathways are known, it may provide insights in managing toxicant-induced Sertoli cell and/or testis injury. In this context, it is of interest to note that the toxicants to be discussed in this review have also been studied extensively in other epithelia and tissues, including their likely mechanisms of action in causing different pathological conditions. For instance, studies have shown that cadmium, a known carcinogen and a toxicant with a relatively long half-life of >20 years [18], causes cancers (in lung, breast, kidney and other organs) via multiple mechanisms, including inhibition of DNA damage repair, induction of oxidative stress, inhibition of apoptosis, and aberrant gene expression [75,76,77]. However, it is not known based on these earlier reports whether cadmium induces disruption of cell-cell anchoring junctions as noted in the testis [39,78]. Even though cadmium has been banned in consumer products, it remains widely used in industry, particularly in the production of nickel-cadmium (Ni-Cd) rechargeable batteries, solar cells, plastic stabilizers and pigments. While the exposure of humans to PFOS and PFOA may not have a causal relationship between cancer [79] and any immune-related health condition [80], the potential risk of human exposure to PFOS/PFOA and neurotoxicity, developmental toxicity (e.g., inducing neonatal mortality) and genetic aberration [81,82,83] have led to the global PFOA ban with exemptions of industrial use. This is due to its thermal and chemical stability, stain resistance, and surfactant nature, making it a key ingredient in fire-fighting foam, hydraulic fluid for aviation and photolithography (https://cen.acs.org/environment/presistent-pollutants/Governments-endorse-global-PFOA-ban/97/web/2019/05 (accessed on 7 December 2021).

## 3. FAK (Focal Adhesion Kinase) and Small GTPase Cdc42

Using different toxicant models, accumulating evidence has suggested that different toxicants, including 2,5-hexanedione, carbendazim [16,20], PFOS [41,84,85] and cadmium [38,86,87], are targeting the actin and microtubule cytoskeletons in the testis. Some of these toxicants, in particular PFOS and cadmium, have shown to exert their disruptive effects through FAK (focal adhesion kinase) signaling (Figure 1 and Figure 2), likely involving small GTPase Cdc42 [88,89], consistent with studies in other epithelia [90,91] and also the mTORC1/rpS6/Akt1/2 signaling complex [13,92].

### 3.1. Focal Adhesion Kinase (FAK)

p-FAK-Y397 and p-FAK-Y407 are the 2 phosphorylated/activated forms of FAK first reported to be expressed in the testis of adult rats in 2003 [52] and 2010 [54], respectively (Figure 1). We provide a critical review on each of these two FAK isoforms regarding their role in regulating spermatogenesis, pertinent to our discussion herein.

#### 3.1.1. p-FAK-Y397

In the rat testis, p-FAK-Y397 is predominantly expressed at the apical ES, at the interface of Sertoli cells and step 8-19 spermatids, surrounding the head of haploid spermatids [52,54], which persists until late stage VIII of the epithelial cycle, just prior to the release of sperms at spermiation [53]. These findings thus suggest that p-FAK-Y397 is crucial to support haploid spermatid adhesion in the seminiferous epithelium during spermiogenesis of the epithelial cycle [17,93]. Indeed, studies in vivo following overexpression of p-FAK-Y397E, the phosphomimetic (and constitutively active) mutant of p-FAK-Y397, in the testis of adult rats was found to delay the release of sperm at spermiation [94]. Furthermore, step 19 spermatids were consistently detected in the seminiferous epithelium, embedded deep inside the epithelium in stage VIII tubules near the basement membrane when spermiation had occurred [94]. Step 19 spermatids were also remarkably noted inside the epithelium even in stage IX tubules, coexisting with step 9 spermatids [94]. This is unusual since these step 19 spermatids should have been differentiated into sperms and be released into the tubule lumen at spermiation at stage VIII, as seen in control testes. This unusual retention of step 19 spermatids that embedded deep inside the seminiferous epithelium in late stage VIII-XI tubules suggest that there were defects in the cytoskeletons, since ES is an actin-rich and MT-dependent anchoring junction. Indeed, detailed examination of the seminiferous epithelium indicated that the actin cytoskeleton surrounding the apical ES, in stage VIII tubules remained uncharacteristically intact [94], unlike control testes when actin cytoskeletons should have undergone degeneration to facilitate the release of sperm. A closer investigation showed that there was a persistent expression of Eps8 (an actin barbed end-capping and bundling protein [95]), thereby maintaining the F-actin network at the site to retain step 19 spermatids in the epithelium [94] when it should have been considerably reduced to facilitate remodeling of the apical ES to support spermiation [17,96]. Furthermore, both nectin 2 (an apical ES adhesion protein expressed by both Sertoli cell and spermatids) and nectin 3 (an apical ES protein expressed only by spermatids) that utilized actin as an attachment site [97,98] were also detected at the apical ES in late stage VIII due to the persistent presence of the F-actin cytoskeletal network [94]. In brief, these findings illustrate that overexpression of p-FAK-Y397E impedes the timely remodeling of the actin cytoskeleton to facilitate the release of sperm at spermiation since the spatio-temporal expression of p-FAK-Y397 at the apical ES is necessary to support haploid spermatid maturation. Yet its persistent presence in late stage VIII tubules (through its overexpression) causes unwanted retention of mature elongated spermatids, leading to defects in spermatogenesis.

#### 3.1.2. p-FAK-Y407

On the other hand, p-FAK-Y407 is also robustly expressed in the rat testis at the apical ES, but unlike p-FAK-Y397, p-FAK-Y407 is also highly expressed at the basal ES/BTB at the Sertoli cell-cell interface, near the basement membrane [54]. Using different phosphomimetic mutants, including both constitutively active and inactive mutants of p-FAK-Y397 and pFAK-Y407, it was shown that p-FAK-Y397 is primarily used to support apical ES, whereas p-FAK-Y407 supports basal ES/BTB function. These two FAK isoforms regulate the corresponding ES function through their effects on actin dynamics, in particular actin polymerization, which in turn modulates actin cytoskeletal organization across the seminiferous epithelium [54]. Studies have shown that primary Sertoli cells cultured in vitro are capable of establishing a functional TJ permeability barrier that mimics the BTB in vivo [33]. However, treatment of Sertoli cells with PFOS (20 µM) in vitro was found to perturb the Sertoli cell TJ permeability barrier function concomitant with extensive disruption of actin filaments across the Sertoli cell cytosol and a down-regulation of p-FAK-Y407 expression [41]. Interestingly, overexpression of p-FAK-Y407E, the phosphomimetic and constitutively active mutant of p-FAK-Y407, in Sertoli cells cultured in vitro was capable of rescuing Sertoli cells from the PFOS-mediated TJ barrier disruption [41]. More important, overexpression of p-FAK-Y407E was capable of rescuing PFOS-induced F-actin disorganization across the Sertoli cell cytosol [41]. This finding has thus unequivocally demonstrated that FAK exerts its effects to support spermatogenesis through cytoskeletal organization. In fact, the use of FAK-specific miR-135b (microRNA-135b, specific to knockdown FAK [99,100]) was found to worsen the PFOS-induced Sertoli cell TJ barrier disruption and also the PFOS-mediated disruptive organization of actin cytoskeleton across the Sertoli cell cytosol [41]. The role of p-FAK-Y397 and p-FAK-Y407 that supports apical and basal ES function is summarized and shown in Figure 3.

#### 3.1.3. Potential Therapeutic Use of p-FAK-Y407E for Management of Toxicant-Induced Male Infertility

An important breakthrough in the study of FAK and its likely impact on male reproductive function in humans came unexpectedly from a study using a human p-FAK-Y407E phosphomimetic (and constitutively active) mutant and primary human Sertoli cells to examine its role in Sertoli cell function in 2017 [85]. Earlier studies have shown that overexpression of rat p-FAK-Y407E mutant in primary cultures of rat Sertoli cells can mitigate the PFOS-induced Sertoli cell injury [41]. For instance, treatment of Sertoli epithelium with an established functional TJ barrier with PFOS (15 µM) induces a transient Sertoli cell TJ permeability barrier disruption, and silencing of FAK by RNAi using a specific FAK miRNA (miR-135b) also worsens the PFOS-mediated Sertoli cell TJ barrier disruption [41]. These observations have been reproduced in studies using human Sertoli cells and a human p-FAK-Y407E mutant [85]. To further expand the earlier findings in rat Sertoli cells, it has been shown that overexpression of human p-FAK-Y407E in human Sertoli cells with an established functional TJ barrier is capable of blocking PFOS (20 µM)-induced F-actin and microtubule cytoskeletal disorganization [85]. These findings thus illustrate that p-FAK-Y407E is not only capable of promoting F-actin organization but also microtubule cytoskeletal organization. It is likely that p-FAK-Y407E exerts its protective effects by promoting the proper distribution of the actin regulatory proteins, namely Eps8 and Arp3, at the human Sertoli cell cortical zone [101]. In this context, it is noted that Eps8 is an actin barbed-end capping and bundling protein [102,103], whereas Arp3, which together Arp2 creates the Arp2/3 complex, is crucial to support branched actin polymerization [103,104]. As such, the combined effects of Eps8 and the Arp2/3 complex are necessary to provide plasticity to the Sertoli cell-cell interacting site (e.g., basal ES) that constitutes the BTB to facilitate continuous remodeling to support the transport of developing preleptotene spermatocytes across the BTB [103]. The actions of Eps8 and the Arp2/3 complex also confer plasticity to the Sertoli-spermatid interacting site (e.g., apical ES) to support the transport of haploid spermatids across the seminiferous epithelium in the adluminal (apical) compartment [103]. Furthermore, overexpression of the human p-FAK-Y407E constitutively active mutant also promotes proper re-distribution of microtubules across the human Sertoli cell cytosol which are disrupted by PFOS, mitigating the disruptive effects of PFOS on Sertoli cell microtubule cytoskeletal organization [85]. This microtubule effect is likely mediated through a re-distribution of the microtubule plus (+) end targeting protein (+TIP) EB1 (end binding protein 1) [85]. Earlier studies have shown that EB1 promotes microtubule stability, preventing microtubules from undergoing shrinkage that leads to microtubule catastrophe [105,106].

#### 3.1.4. Additional Remarks—Possible Involvement of Akt1/2 Activation

Studies have shown that FAK is typically considered an upstream signaling protein of Akt, most notably during pathogenesis, such as cancer metastasis in colon and prostate cancers [107,108,109]. It was also shown that treatment of primary rat Sertoli cells by PFOS (20 or 50 µM) also induced a considerable down-regulation of p-Akt1/2, most notably p-Akt1-T308, p-Akt1-S473 and p-Akt2-S474 [84]. However, the use of SC79 (2-amino-6-chloro-α-cyano-3- (ethoxycarbonyl)-4H-1-benzopyran-4-acetic acid ethyl ester, Mr 364.78) is capable of mitigating the PFOS-induced Sertoli cell injury [84]. This observation is important since SC79 is a specific p-Akt1/2 activator that is known to bind to the pleckstrin homology (PH) domain of Akt, mimicking the binding of PtdIns (3,4,5)P3 to activate Akt by inducing conformational change, which in turn enhances phosphorylation at the p-Akt1-T308 and p-Akt1-S473 sites [110]. Furthermore, the disruptive effects induced by PFOS on rat Sertoli cells [84] regarding disruption of the cytoskeletal organization of both the actin and microtubule cytoskeletons are similar to that noted in human Sertoli cells [85]. The use of SC79 promotes the proper organization of these two cytoskeletons by rescuing Sertoli cells from the PFOS-mediated injury [84]. Additionally, the use of SC79 also rescues Sertoli cell injury induced by PFOS regarding disruptive changes in the localization of TJ (e.g., CAR, ZO-1) and basal ES (*N*-cadherin, β-catenin) proteins, and also the corresponding actin (actin bundling protein palladin, branched actin nucleation protein Arp3, and p-FAK-Y407) and microtubule (e.g., EB1, detyrosinated α-tubulin) regulatory proteins (or its more stabilized isoform) at the Sertoli cell-cell interface [84]. In brief, SC79 is capable of restoring PFOS-induced Sertoli cell injury through its effects on both actin and microtubule cytoskeletons, analogous to the use of p-FAK-Y407E for its overexpression in Sertoli cells. Taken collectively, these studies have demonstrated unequivocally that both FAK and its downstream signaling partner, p-Akt1/2, are two involving non-receptor protein kinases in the signaling cascade mediated by PFOS to perturb cell-cell interactions between testicular cells during male reproductive dysfunction. These findings are summarized and shown in Figure 2. Additionally, these findings provide an important framework to take these studies to a translational path to investigate the possibility of using this approach to manage male reproductive dysfunction, including infertility, in particular, if toxicants are involved in the etiology.

### 3.2. Small GTPase Cdc42

Studies have shown that Cdc42, a member of the Rho GTPase family, which together with other family members, including RhoA, Rac1, Rac2, RhoH, RohD/F, RhoU/V affect cell movement, endocytosis, cell morphology and cell cycle progression through their effects on actin cytoskeletal organization [111,112,113,114,115]. More important, Cdc42, together with Rac1 and RhoA, affect the dynamics of filopodia, lamellipodia and stress fibers, namely their assembly (formation), disassembly and maintenance [116,117,118,119,120]. The concerted efforts of these GTPases thus confer cell migration in fibroblasts, macrophages, and other locomotive cells under physiological conditions, including cancer cells during tumorigenesis, making GTPases one of the prime targets of cancer treatment. On the other hand, Cdc42 is an emerging downstream modulator of FAK by regulating the cytoskeletal organization of actin and possibly microtubules [121,122,123]. In fact, Cdc42 is likely working in concert with FAK to support cytoskeletal organization in Sertoli cells in response to the epithelial cycle of spermatogenesis based on studies using the NC1-peptide model [88] and the TGF-ß3 model [89]. Studies have shown that NC1 peptide is released from the structural collagen α3 (IV) chains in the basement membrane, which in turn, serves as a biologically active peptide to induce Sertoli cell BTB remodeling, thereby facilitating the transport of preleptotene spermatocytes across the BTB at stage VIII-early stage IX of the epithelial cycle [124]. In brief, the NC1-peptide that induces Sertoli cell BTB remodeling, such as by perturbing the Sertoli cell-TJ permeability barrier function following its overexpression in Sertoli cells, is mediated through changes in the organization of both actin and microtubule cytoskeletons [124,125]. Additionally, these effects on cytoskeletal organization are mediated through activation of Cdc42, but not RhoA [88]. Overexpression of a Cdc42-T17N dominant negative mutant in Sertoli cells cultured in vitro, via a single mutation of amino acid residue 17 from Thr (T) to Asn (N) by site-directed mutagenesis, making this mutant constitutively inactive [89], is able to abolish the disruptive effects of NC1-peptide on Sertoli cell-TJ barrier function [88]. More importantly, a recent report has shown that NC1-peptide-induced BTB disruption and defects in spermatogenesis in the testis in vivo following its overexpression in the testis are also associated with a considerable down-regulation of p-FAK-Y397 and p-FAK-Y407 [126]. Taken collectively, these findings have thus demonstrated unequivocally that the NC1-peptide-induced effects on spermatogenesis involves Cdc42 activation and p-FAK-Y407 down-regulation, suggesting that FAK and Cdc42 are two signaling proteins that work in concert to modulate testis function. Other recent reports have also shown that Cdc42 is essential to support spermatogenesis. First, Cdc42 expressed by Sertoli cells is required for male germline niche development in mice [127]. It was shown that Sertoli cell-specific Cdc42-deficient mice failed to sustain germline niche development, likely due to a down-regulation of GDNF [a critical factor known to support spermatogonial stem cell (SSC) maintenance], DMRT1 and SOX9 (both genes are necessary to support Sertoli cell development) and a concomitant reduced MAPK1/3 expression in the Sertoli cell nucleus [127]. Collectively, these data suggest that Sertoli cell Cdc42 is essential for germline niche function via MAPK1/3-dependent GDNF expression [127]. Second, conditional deletion of Cdc42 in Sertoli cells also led to a loss of Sertoli cell polarity, an increase in apoptosis, and round spermatids, which failed to develop to elongated spermatids through spermiogenesis due to Sertoli cell defects [128], illustrating the pivotal role of Cdc42 in supporting spermatogenesis. This latter finding is important since studies have shown that proteins that support cell polarity, such as the Par-, the Crumbs-, and the Scribble-based polarity complexes all exert their effects through cytoskeletons [129,130]. Collectively, these findings thus support the notion that Cdc42 likely mediates its effects through changes in cytoskeletal organization, including its role at the germline niche.

## 4. Concluding Remarks and Future Perspectives

As discussed above, it is increasingly clear that cell-cell interactions between testicular cells in the testis can induce activation of several signaling proteins, most notably FAK and its downstream signaling partner Ak1/2 (Figure 2), to support spermatogenesis based on studies of toxicant and pharmaceutical models (Table 1). Importantly, some of these studies performed earlier in rodents have been reproduced and expanded in primary cultures of human Sertoli cells, making these findings more clinically relevant. The immediate step in the near future is to move these studies to a translation path so that these findings can be carefully evaluated using a therapeutic approach. Furthermore, research should also be expanded to develop a new approach to target these reagents, either the plasmid DNA (e.g., pcDNA 3.1 (+)/FAK-Y407E mutant) used for transfection or the activator of p-Akt1/2, directly to the testis in order to reduce any unwanted side effects, if any, in unintended organs/tissues. The use of nanoparticle-based technology should be considered in future investigations, such as the use of an FSH-based approach since Sertoli cells exclusively express FSH receptors in the body of human males.

In this context, it is also noteworthy to mention the possible effects of pollutants (e.g., heavy metals including chromium, copper) that induce molecular alterations of sperm nuclear basic proteins (SNBP) and DNA damage through alterations in protamines/histones ratio and oxidative DNA damage, as well as changes in sperm protamine-like proteins [131,132], which in turn induce transgenerational inherited defects in humans. Besides these studies in humans, sub-toxic doses of cadmium (at 5 µM) were also found to induce alterations of sperm protamine-like proteins in mussels, which are the major basic nuclear component of sperm chromatin, affecting chromatin organization of spermatozoa [133], similar to studies in humans. Another heavy metal, mercury (Hg), at 1, 10 and 100 pM as HgCl_2_, was also found to induce alterations of protamine-like proteins that impeded sperm chromatin organization in mussels, causing DNA damage [134,135]. Furthermore, oxidative stress, such as that mediated by exposure of humans to environmental toxicants/pollutants, in particular heavy metals, are also known to impede spermatogenesis and human sperm metabolism and apoptosis [136,137]. At present, it is not known if the toxicant-induced epigenetic and transgenerational reprogramming of reproductive function, or oxidative mediated DNA damages, involve disruptive changes in signaling cascade. This possibility must be carefully evaluated in future studies.

In summary, this review provides a timely evaluation of how environmental toxicants may impede male infertility through changes in signaling cascades/pathways, providing a fresh view on the worldwide declining male fertility in countries across the globe [138,139,140].

**Table 1:** Effects of CdCl_2_ and PFOS on testis and Sertoli cell function *.

Toxicant	Species	Tissue/Cell	Doses/Route	Observed Effects	Reference
Cadmium Chloride (CdCl_2_)	Rat	Testis	3 mg/kg b.w., i.p.	Loss of occludin at the BTB in the epithelium	[32]
	Rat	Testis	3 mg/kg b.w., i.p.	Changes in spatial distribution of MAPs (MAP1a and CAMSAP2) in the seminiferous epithelium	[87]
	Rat	Testis	3 mg/kg b.w., i.p.	CdCl_2_-induced BTB disruption, an increase in TGF-β2 and TGF-β3 (but not TGF-β1) and p-p38 -MAPK, a down-regulation of occludin and ZO-1	[78]
	Rat	Testis	3 mg/kg b.w., i.p.	Down-regulates the expression of efflux (e.g., P-glycoprotein, Mrp1, Abcg1) and influx (e.g., Oatp3, Slc15a1, Scl39a8) drug transporters	[141]
	Mouse	Testis	2 mg/kg b.w., i.p.	Induces germ cell apoptosis in testes	[142]
	Rat	Testis	2 mg/kg b.w., i.p.	Reduces body weight and testes weight, increases malondialdehyde content, reduces superoxide dismutase, glutathione peroxidase, catalase, and glutathione contents	[143]
	Rat	Testis	3 mg/kg b.w., i.p.	Induces epithelial damage (e.g., edema), disorganization of collagen fibers, microvascular damage	[144]
	Rat	Sertoli Cell	3 μM	Perturbs TJ barrier, induces occludin endocytosis in parallel with FAK and ZO-1	[38]
	Rat	Sertoli Cell	5–10 μM	Perturbs TJ assembly dose-dependently without any apparent cytotoxicity	[40]
	Rat	Sertoli Cell	0.1–5 μM	Perturbs Sertoli cell TJ barrier dose dependently	[40]
	Human	Human Sertoli cell	0.5–20 μM	Induces truncation actin filaments via disruptive distribution of Eps8 and Arp3	[86]
Perfluoro-octanesulfonate (PFOS)	Rat	Sertoli Cell	10–20 μM	Induces Sertoli cell TJ barrier disruption mediated by a reduced expression of p-FAK-Tyr407 and Cx43, F-actin disorganization and impaired GJ intercellular communication, mislocalization of proteins at the cell-cell interface	[41]
	Rat	Sertoli Cell	10, 20, 50 μM	Induces Sertoli cell injury by perturbing TJ barrier, disorganization of actin cytoskeleton due to mis-localization of Arp3 and palladin, mis-distribution of BTB-associated proteins, downregulation of p-Akt1-S473 and p-Akt2-S474.	[84]
	Rat	Sertoli Cell	20–40 μM	Induces Sertoli cell injury through truncation of actin filaments and MTs, which can be rescued by overexpressing p-FAK-Y407E mutant	[84]
	Rat	Sertoli Cell	20 μM	Perturbs Sertoli cell TJ barrier, causing disruption of actin filaments in cell cytosol, perturbing the localization of cell junction proteins, reducing expression of GJ protein Cx43	[145]
	Rat	Sertoli Cell/Gonocyte Cocultures	0, 1, 10, 50, and 100 μM	Reduces cell viability, induces reactive oxygen species (ROS) production dose-dependently and disrupts organization of vimentin and actin filaments	[146]
	Mouse	Testis Sertoli Cell	0.25–50 mg/kg/day (oral gavage) 10–30 μM	Reduces sperm count, induces Sertoli cell injury via an increase in vacuolization in Sertoli cells in seminiferous epithelium, disruptive changes in BTB ultrastructure leading to disassembly based on studies in vivo; perturbs Sertoli TJ barrier function, induces mis-distribution of BTB-associated proteins at the cell-cell interface, and increases expression of activated p38-MAPK and Erk1/2	[37]

* This table is not intended to be exhaustive. It contains several selected recent reports to illustrate intercellular junctions are the target of environmental toxicants using cadmium and PFOS as study models. References from many investigators could not be cited due to space limitations.
